# Bad research is not all bad

**DOI:** 10.1186/s13063-023-07706-1

**Published:** 2023-10-20

**Authors:** Fergus Hamilton, David Arnold, Richard Lilford

**Affiliations:** 1 Infection Science, Southmead Hospital, North Bristol NHS Trust, Bristol, BS10 5NB UK; 2 MRC Integrative Epidemiology Unit, Oakfield House, University of Bristol, Bristol, BS10 5NB UK; 3 Academic Respiratory Unit, Southmead Hospital, North Bristol NHS Trust, Bristol, BS10 5NB UK; 4 NIHR ARC West Midlands, Institute of Applied Health Research, College of Medical and Dental Sciences, University of Birmingham, Edgbaston, Birmingham, B15 2TT UK

**Keywords:** Trials, Evidence, Policy

## Abstract

In this commentary, we discuss a recent article in *Trials* that raised concerns about the number of poorly performed randomised trials in the medical literature and discuss the trials literature more widely. Although we all aim for higher methodological standards in trials, we argue that (i) the idea that ‘most randomised trials are bad’, which the recent article concludes is an overly simplistic representation of the situation, and (ii) the suggestion that an increased focus on methodological review during trial development (e.g. ethical boards performing some assessment of the methodologists on a trial), while well meaning, may have negative unintended consequences. We therefore propose that (a) trials should be assessed on their merits and weaknesses, including an assessment of risk of bias but placing that in a wider context; (b) we should recognise that although the methodological conduct of trials is of utmost importance, interventions that aim to improve this could have unintended consequences—such as bureaucracy—that have an overall negative effect; and (c) we should therefore generate an evidence base for policy interventions to improve conduct of trials rather than applying arbitrary rules.

## Background

In a recent article in *Trials*, Pirosca and colleagues wrote about the continuing scandal of bad research [1], echoing Doug Altman’s views of the ‘scandal of poor medical research’, nearly 30 years later [2]. In their analysis, they utilised data from Cochrane Collaboration reviews to estimate that more than half (56%) of all randomised trials included in these Cochrane reviews were ‘bad’. To define ‘bad’, they took the evidence from Cochrane reviewers, who assess all trials as either low or high (or unclear) risk of bias on a number of domains, and then give an overall assessment. The Cochrane view, as expressed in the handbook, is that if one domain is high risk, then the whole trial is high risk [3].

Pirosca and colleagues’ view is that if a trial is at high risk of bias (by definition above, even if only one domain), then this is a ‘bad trial’. They go on to make estimations of the cost of these trials (ranging from £726 million to £8 billion) and describe a set of proposals in order to remediate this. We absolutely share Pirosca and colleagues (and the late Doug Altman’s) view on the scandal of poor research and recognise ongoing challenges with poor trials but feel claim that > 50% of the randomised trial literature as ‘bad’ and ‘[trials] we have little confidence in’ is unhelpful and is too simplistic a view. We argue trials should be evaluated with more judgement and without applying rules to dichotomise evidence. In addition, although we support many of their proposals (increased funding for methodologists, greater focus on methods), some of their proposals such as mandating funders and ethics boards review the methodological make-up of a trial team may—despite being well meaning - not add benefit [4].

## Main

It is important to recognise that randomised trials are hard to do, for numerous reasons: ethical, logistical, financial, and practical. Additionally, as anyone who has sat on a funding panel can attest to, even seemingly simple questions (‘how should we measure this outcome’) can divide expert methodologists, clinicians, and patients. At every stage of a trial, researchers must weigh up opportunity costs, direct costs, pragmatism, and many other factors. It is well recognised that one of the major challenges to trials is research bureaucracy [4–10]. As such, we should recognise (as one recent article is entitled) that well-intentioned policy can have unintended consequences [4].

To explore our argument that binary assessment of trials is too simplistic, we focussed on four reviews comprised of ‘bad’ trials identified in Pirosca et al. We purposively sampled trials from infection/public health—our speciality.

The first review, in which every trial was considered bad, was a review of house modifications (e.g. screening doors) to prevent malaria (*n* = 2 studies) [11]. Both trials suggested (with a degree of uncertainty) some benefit. Both were considered high risk of bias (and therefore ‘bad’) because of participants were not blinded/masked. Given these are cluster randomised trials, blinding would be impossible to achieve (a point noted by the Cochrane reviewers). Additionally, the Cochrane reviewers did not feel the statistical analysis in either trial was appropriate (improperly accounting for clustering), rating this again as a high risk of bias. We note the statistician who ran the analyses on one of the trials is a Professor of Epidemiology and Biostatistics and expert in malaria at the London School of Tropical and Hygiene Medicine. We state this not to claim that the trial was analysed correctly but simply that experienced methodologists can and often do disagree on the appropriateness of any given analysis, which makes the application of a simple rule that a trial is deemed high risk of bias because the reviewers disagreed with the analytical choices challenging.

The second review focussed on hydroxychloroquine (or chloroquine) to prevent COVID-19 (*n* = 14 studies) [12]. All trials except one were recorded as high risk of bias. The one trial recorded as unclear risk of bias was the RECOVERY trial [13] (although this may be an error, it is recorded as low risk of bias in the original Cochrane review). Despite the fact that nearly all these trials were ‘bad’, the review was able to conclude (correctly, if we are to trust RECOVERY) that hydroxychloroquine has no place in the management of COVID-19. Broadly speaking, the trials excluding RECOVERY had similar effect estimates to RECOVERY and provide useful confirmatory evidence. Although these trials are not perfect, it is clear that they have contributed to the evidence and furthered policy.

Finally, we look at acute respiratory infection, where the two Cochrane reviews detailed in Pirosca et al. focussed on the use of rapid antigen tests in sore throat to guide antibiotic prescribing [14] and the role of antibiotics vs no antibiotics for non-severe childhood pneumonia [15]. In the first review, all five trials were at high risk of bias because participants and clinicians were unblinded. Given that the review question was whether rapid antigen testing reduced prescribing, it is hard to imagine how the trial could have been performed blinded. In the second review (on childhood pneumonia), one out of the three trials was considered high risk of bias, because, despite adequate blinding of clinicians, patients, and researchers, the trial statistician was unblinded [16]. Given there is ongoing discussion by triallists about the risks and benefits of blinding statisticians, we would argue this may well have been the correct decision and was highly unlikely to bias the trial [17, 18]. The trial (*n* = 1199) concluded that placebo was inferior to amoxicillin (adjusted relative risk of treatment failure, 1.78; 95% CI, 1.07–2.97%) on one outcome of interest. We find it hard to believe that clinicians who practice in this field would think this trial ‘bad’ and would not consider the evidence from it when treating a child with non-severe pneumonia.

The point we are trying to make is that on closer review of a number of these trials, they are clearly not bad trials. They may not be perfect trials, and others may disagree on how they were performed or analysed, and they may have higher risk of bias than other trials, but they are clearly not research waste or useless for decision making. Many were published by research groups with great expertise in trial design and funders that have stringent methodological review. Moreover, careful consideration of potential bias can prompt further considerations. Some potential biases are plausible in one direction only and some may lend themselves to sensitivity analysis. Turner and Spiegelhalter have suggested specifying a probability density for the magnitude of any plausible bias [19]. Nuanced judgements and principled exploratory analyses are swept aside by rigorous application of rules. An illustrative example of these rules can be seen in the 2017 Cochrane review of direct acting antivirals (DAAs) for hepatitis C [20]. These drugs have revolutionised the management of hepatitis C and alongside active case finding are likely to lead to elimination of hepatitis C in the UK by 2030 and within decades worldwide as they lead to an approximately 97% cure rate [21–25]. However, the Cochrane review identified that all randomised trials were at high risk of bias and concluded (in part due to this risk of bias assessment) that the review could not ‘confirm nor reject that DAAs had any clinical effects’ [on hepatitis C] [20]. This was widely criticised by multiple experts and clinicians as an inappropriate interpretation of the evidence, and DAAs remain the current standard of care in hepatitis C by the World Health Organisation [26], the National Institute for Health and Care Excellence [27], and many other guidance bodies, and we do not think anyone seriously doubts their efficacy [24, 28–30]. We therefore suggest that the assessment of trials performed by Pirosca et al. is incomplete, and that claiming that trials that are at high risk of bias are ‘bad’ and that we have ‘little confidence in’ is unfair. These trials may not be perfect, but it is clear that in many circumstances the evidence gained from them is useful. All trials—even those at low risk of bias—require interpretation in line with other evidence (e.g. triangulation [31]), and we do not support the view that 50% of trials are ‘bad’, while accepting that we should aim to improve methodological quality wherever we can.

We therefore turn to the second question: how do we improve methodological quality of trials? We focus here on the UK, where we are based, but our arguments likely apply elsewhere. Pirosca and colleagues suggest a number of policy recommendations which we agree with (increased funding for methodology and increased methodologists). However, we disagree with their first two recommendations—mandating funders and ethical boards review the methodological experience of trial teams—and are sceptical of the third (risk of bias tool mandated). Firstly, no policy intervention comes free of unintended harmful effects and costs [4, 8]. For example, a policy introduced to reduce in ‘time to first dose’ of antibiotic from 8 to 4 h in community-acquired pneumonia in line with guidance likely led to an increase in diagnostic error [32]. This cost was compounded by subsequent trial evidence showing limited benefit of earlier dosing in critically ill patients with infection, suggesting the policy may likely have led to net harm [33].

Therefore, it is important to evaluate exactly how Pirosca et al. would propose this occurs. If the requirement is simply to have a ‘named’ methodologist, then this approach would have little cost (apart from another online tick box), but almost no benefit, as one of the triallists would just be named the methodologist at application. If the requirement is that the named methodologist is somehow assessed, this now creates a large number of costs: who does the assessment? How are they assessed? What if there is a disagreement? One of the authors of this article (RJL) has been running randomised trials for > 30 years, published widely in trial methodology but is a clinician whose title is Professor of Public Health and who has no formal methodological qualifications. Is he a methodologist? To ascertain this, this would require funders and ethics boards to search for the methodologist, identify relevant outputs, assess them (ideally in duplicate, etc. so as to avoid bias), and make a judgement. Of course, some of this ‘cost’ could be placed on to the researcher, who would have to fill in another form at the time of application and ethical approval, which is exactly the kind of bureaucracy that is hampering the conduct of randomised trials today [34]. This cost will be multiplied by deciding who has the ability to assess the assessor and other associated costs. We do not make the argument that trial design should be a free-for-all, but simply that all policy interventions have costs, and that an assessment of trial methodology should be performed on the trial itself, rather than adding binary rules about who and who cannot perform a trial.

The appropriate judgement of the methodology of an RCT should be on its methodology, not on whether there is an author who is named as a methodologist. We should recognise that the continued existence of poor methodological approaches in trials is a complex problem that is unlikely to be solved (without cost) by simple interventions, while there are other important issues in the conduct of randomised trials that must also be considered.

We therefore propose that (a) trials should be assessed on their merits and weaknesses, including an assessment of risk of bias but placing that in a wider context; (b) we should generate an evidence base for policy interventions to improve conduct of trials; and (c) we should recognise that although the methodological conduct of trials is of utmost importance, interventions that aim to improve this could have unintended consequences—such as bureaucracy—that have an overall negative effect.

## Data Availability

NA.
